# International Clinics

**Published:** 1894-06

**Authors:** 


International Clinics.—Vol. 1. Fourth Series. 1894. J. B. Lippincott Company.

The profession will welcome this, the first volume of another series of entertaining and instructive lectures. The first place in the work is fittingly occupied by a cut of the great Charcot, with a memorial of his life and work by M. Allen Starr. Space would forbid even the enumeration of the contributions, but as usual they have been carefully selected from the lectures of the best teachers of both America and Europe, and each and every lecture will be of interest to both the student and practitioner.

